# Development of a rapid and simple tetracycline detection system based on metal-enhanced fluorescence by europium-doped AgNP@SiO_2_ core–shell nanoparticles†

**DOI:** 10.1039/c8ra03185a

**Published:** 2018-07-05

**Authors:** Pei Li, Sathish Kumar, Ki Soo Park, Hyun Gyu Park

**Affiliations:** Department of Chemical and Biomolecular Engineering (BK21+ Program), KAIST 291 Daehak-ro, Yuseong-gu Daejeon 34141 Republic of Korea hgpark@kaist.ac.kr +82-42-350-3910 +82-42-350-3932; Department of Biological Engineering, College of Engineering, Konkuk University Seoul 05029 Republic of Korea kskonkuk@gmail.com +82-2-450-3742 +82-2-450-3742

## Abstract

We herein describe a rapid and selective sensing platform for tetracycline (Tc), which relies on the metal-enhanced fluorescence (MEF) effect of europium (Eu^3+^)-doped silver-silica core–shell nanoparticles (AgNP@SiO_2_). The developed assay utilizes AgNP@SiO_2_ as a key detection component, which is systematically optimized to have a silica shell thickness suitable for the effective MEF phenomenon. In principle, the AgNP@SiO_2_, which binds to Eu^3+^ through the electrostatic interaction, captures Tc by selective chelation with Eu^3+^, leading to significant fluorescence enhancement of the EuTc complex. Based on this novel strategy, we determined Tc as low as 83.1 nM with a total assay time of less than 10 min, which is comparable to or better than that of the previous fluorescence-based methods. Furthermore, the practical applicability of this strategy was successfully demonstrated by detecting Tc in tap water. This work highlights the unique features of AgNP@SiO_2_ for MEF-based biosensing applications.

## Abstract 2

Tetracycline (Tc) selectively binds with europium (Eu^3+^) which emits red fluorescence (615 nm) upon excitation at 400 nm.

## Graphical abstract

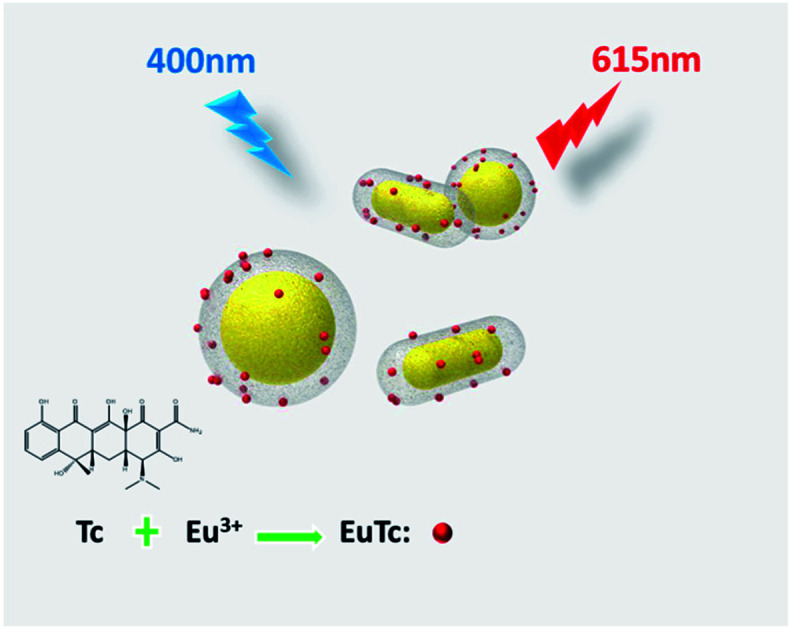

## Introduction

Tetracycline (Tc) is a versatile antibiotic against a broad range of pathogens, which is known to inhibit protein synthesis by binding to a small 30S ribosomal subunit and preventing the attachment of aminoacyl-tRNA to the ribosomal acceptor site.^1,2^ However, Tc, a life-saving molecule, has been increasingly over- and mis-used, which results in the emergence of antimicrobial resistance.^3^ In addition, ingested Tc causes side effects such as nail discoloration and onycholysis in hypersensitive individuals.^4^ Therefore, a great interest has been aroused for the rapid and simple detection of Tc.

Until now, various methods have been utilized to determine Tc, including liquid chromatography/mass spectrometry, high-performance liquid chromatography,^5,6^ capillary electrophoresis,^7^ and chemiluminescence.^8,9^ Especially, novel electrochemical and colorimetric detection assays based on DNA aptamers specific to Tc have been intensively attempted due to their good selectivity and sensitivity for Tc detection.^10,11^ However, they entail complicated preparation steps and long assay procedures, which have limited their practical applications.

In this work, for the development of a rapid, simple, and selective sensing platform for Tc, we employed europium (Eu^3+^) which belongs to the lanthanide ions with the unique features, including sharply spiked emission peak (<10 nm full width at half maximum), large Stokes shift (>150 nm), high quantum yield, and long decay times on the μs/ms timescale, much longer than the ns timescale of most biological autofluorescence.^12,13^ Based on the fact that Eu^3+^ can chelate Tc with a β-diketonate structure to form a stable fluorescent complex (EuTc),^14–16^ we adopted the metal enhanced fluorescence (MEF), a phenomenon that occurs when a fluorophore is at the adjacent place of the metallic surface, resulting in the increased fluorescence intensity, photostability, and radiative decay rates, in order to significantly increase the fluorescence signal of EuTc complex.^17–20^ Specifically, we synthesized AgNP@SiO_2_ with the optimal silica shell thickness, which binds to Eu^3+^ through the electrostatic interaction, and promotes the effective MEF phenomenon. As a result, the presence of Tc, which chelates Eu^3+^ to form the stable EuTc complex on the surface of AgNP@SiO_2_, produces the significantly increased red fluorescence. With this novel strategy, we successfully analyzed Tc with the high sensitivity and selectivity even in tap water.

## Experimental

### Chemicals and materials

Sodium citrate tribasic dehydrate, poly(sodium styrene sulphonate) (PSSS, 1,000 kDa), sodium borohydride (NaBH_4_), tetraethylorthosilicate (TEOS, 98%), tetracycline hydrate (Tc, 99%), europium(iii) nitrate pentahydrate (99.9%), sodium nitrate (NaNO_3_), potassium nitrate (KNO_3_), magnesium nitrate hexahydrate (Mg(NO_3_)_2_·6H_2_O), cobalt(ii) nitrate hexahydrate (Co(NO_3_)_2_·6H_2_O), lead(ii) nitrate (Pb(NO_3_)_2_), calcium nitrate tetrahydrate (Ca(NO_3_)_2_·4H_2_O), aluminum nitrate nonahydrate (Al(NO_3_)_3_·9H_2_O), iron(iii) nitrate nonahydrate (Fe(NO_3_)_3_·9H_2_O), mercury(ii) nitrate monohydrate (Hg(NO_3_)_2_·H_2_O), copper(ii) nitrate trihydrate (Cu(NO_3_)_2_·3H_2_O), arginine (l-) (Arg), l-aspartic acid (Asp), l-histidine (His), l-lysine monohydrochloride (Lys), l-cysteine hydrochloride monohydrate (Cys), glutathione (GSH), l-ascorbic acid and d-(+)-glucose were purchased from Sigma-Aldrich. Silver nitrate (AgNO_3_) and methylamine (MA, 40% solution in water) were obtained from Kojima Chemicals Co., LTD. and Junsei Chemical Co., Ltd., respectively. All of the solvents and reagents were used without any further purification.

### Preparation of silver nanocore

The large AgNPs were synthesized according to the previously reported method.^17^ In brief, AgNPs were prepared by adding 4 mL of sodium citrate aqueous solution (38.8 mM) into 196 mL of boiling AgNO_3_ (1.05 mM) under vigorous stirring. The color of the solution turned from colorless to yellow, and finally turned to gray-yellow within 4 min. After boiling for 20 min, the reaction solution was cooled to room temperature under vigorous stirring. The as-prepared large AgNPs were stored at 4 °C. In addition, the small AgNPs were synthesized according to the previously reported method.^21^ In brief, 5 mL of aqueous sodium citrate solution (2.5 mM), 0.25 mL of aqueous poly(sodium styrene sulphonate) (PSSS) (500 nM) and 0.3 mL of freshly prepared sodium borohydride (NaBH_4_) (10 mM) were mixed. Then, 5 mL of AgNO_3_ (0.5 mM) was added dropwise to this solution at the rate of 2 mL min^−1^ under vigorous stirring, which was stirred for further 3 min. The as-prepared small AgNPs were stored at 4 °C.

### Synthesis of silica shells on silver nanocore

The silver nanoparticles were coated with silica shells according to a modified Stöber method.^17,21^ Under vigorous stirring, 3.6 mL of the as-prepared AgNP solution was mixed with 32.08 mL of ethanol, 3 mL of deionized water and 200 μL of tetraethoxysilane (TEOS) at different concentrations (200 mM, 400 mM, 600 mM and 800 mM). The final concentrations of TEOS in each suspension are 1 mM, 2 mM, 3 mM and 4 mM, respectively. The silica coating was initiated by the dropwise injection of 1.12 mL methylamine (MA, 40% in water) to the suspension. The final concentration of MA is 0.64 mM. The solutions were stirred at room temperature for 30 minutes and then allowed to age without agitation at 4 °C overnight (more than 6 hours). Each suspension of silica-coated silver nanoparticles (AgNP@SiO_2_) was washed with distilled water (D.W.) and ethanol mixture (5 : 4) in a 50 mL tube and centrifuged at 3500 rpm for 35 minutes. The core–shell nanoparticles with the different thickness were collected from the bottom of the 50 mL tube and transferred to a 1.5 mL tube, followed by washing and centrifugation (3500 rpm, 15 minutes) twice with D.W. Finally, the mixture was suspended in D.W.

The concentration of AgNP@SiO_2_ was determined according to Beer Lambert's law *A* = *εcb* (*A*: absorbance, *ε*: molar extinction coefficient, *c*: molar concentration, and *b*: path length). The absorbance spectra of 200 μL of AgNP@SiO_2_ were measured in 96 well plate. The concentration of AgNP@SiO_2_ was calculated by using the parameters: molar extinction coefficient of AgNP@SiO_2_ is 739 × 10^8^ M^−1^ cm^−1^ (ref. 22) and the path length is 0.56 cm.

### Optimization of the reaction conditions

To investigate the impact of the silica shell thickness on the fluorescence of EuTc, 24 μL of Eu^3+^ (100 μM), 12 μL of Tc (100 μM) and 20 μL of AgNP@SiO_2_ (0.133 nM) with the different thickness were added to each tube to make the final volume of each solution to 200 μL. The mixtures were incubated for 5 minutes and the emission spectra were then recorded at the excitation wavelength of 400 nm.

The Eu^3+^ concentration was optimized by adding 0 μL, 6 μL, 12 μL, 24 μL, 36 μL and 48 μL of Eu^3+^ (100 μM) into the solutions containing Tc (6 μM) and AgNP@SiO_2_ (0.02 nM) in a total volume of 200 μL, respectively. The mixtures were incubated for 5 minutes and the fluorescent intensity at 615 nm were then recorded at the excitation wavelength of 400 nm.

The impact of AgNP@SiO_2_ concentration on the fluorescence of EuTc was studied by adding various concentrations of AgNP@SiO_2_ (0.133 nM) in each of the 200 μL solutions containing Eu^3+^ (12 μM) and Tc (6 μM).

### Tc detection procedure

For the detection of Tc, 24 μL of Eu^3+^ (100 μM) and 0 μL, 0.02 μL, 0.2 μL, 2 μL, 6 μL, 12 μL, 24 μL, 36 μL and 48 μL of Tc (100 μM) at different concentrations were incubated for 5 minutes, and 30 μL AgNP@SiO_2_ (0.133 nM) were incubated for 4 minutes and the resulting fluorescence signal was measured on 384 well plate at the excitation and emission wavelengths of 400 nm and 615 nm, respectively.

### Detection of Tc in real samples

The tap water was obtained in the lab and used without further purification. Tc at varying concentrations was spiked into the tap water, which was subsequently analyzed using the same procedure to detect Tc as described above.^23^

### Instruments

Transmission electron microscopy (TEM) images were recorded with a Cs-corrected STEM (JEM-ARM200F). The EuTc-doped AgNP@SiO_2_ samples were cast onto the copper grids (300 mesh) with a lacey carbon film (LC300-CU, Electron Microscopy Sciences) and dried at room temperature overnight. The average shell thickness and the distribution of the SiO_2_ shell thickness were analyzed based on the TEM images. The nanoparticle size distribution was analyzed based on at least 150 nanoparticles by using ImageJ software. Energy dispersive X-ray spectroscopy (EDS) images and element mapping data were also obtained during the TEM measurements. The UV-vis absorption spectra and fluorescence spectra were obtained with an Infinite M200 PRO Multi-Detection Microplate Reader (Tecan).

## Results and discussion

**Scheme 1:**
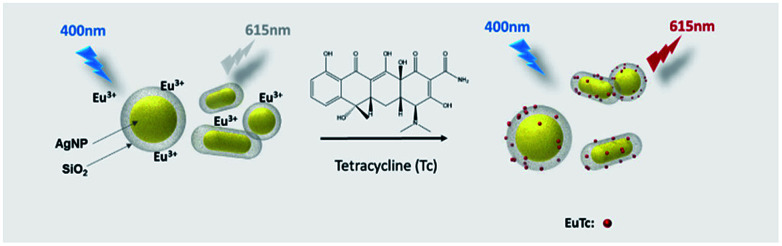
Schematic illustration of the detection of Tc using Eu^3+^-doped AgNP@SiO_2_.

The basic principle of the new strategy to determine Tc is illustrated in Scheme 1, wherein Eu^3+^-doped AgNP@SiO_2_ is utilized as the key detection component. In the absence of Tc, Eu^3+^-doped AgNP@SiO_2_ emits negligible fluorescence upon excitation at 400 nm. On the contrary, in the presence of Tc, Eu^3+^ on the surface of AgNP@SiO_2_ chelates Tc to form a stable EuTc complex. When excited at 400 nm, the EuTc complex emits the significantly increased red fluorescence at 615 nm through the effective energy transfer from Tc to Eu^3+^ and the MEF effect of AgNP@SiO_2_. As a result, Tc could be sensitively determined by measuring the fluorescence signal of the samples.

**Fig. 1:**
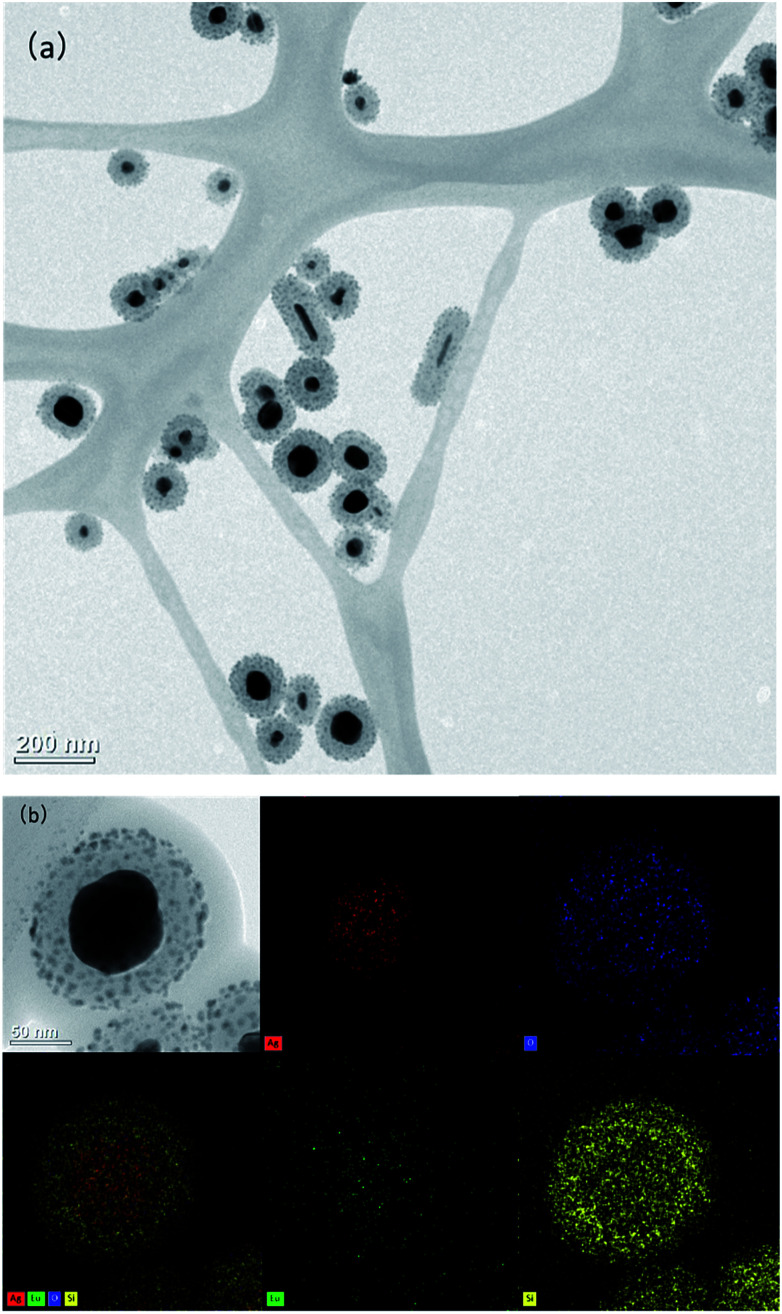
(a) TEM image of Eu^3+^-doped AgNP@SiO_2_. (b) Energy-dispersive X-ray spectroscopy (EDS) image of EuTc-doped AgNP@SiO_2_.

First, we synthesized the AgNP@SiO_2_ by utilizing silica shell as the distance adjusting layer between the fluorescent EuTc complex and the silver core. The core–shell AgNP@SiO_2_ was prepared by following a modified Stöber method (see materials and methods for details). The synthesized AgNP@SiO_2_ were characterized by transmission electron microscopy (TEM). As shown in Fig. 1(a), the silica shell is homogeneously formed around the silver core and Eu^3+^ was doped on the surface of the silica shell, which was confirmed by the energy-dispersive X-ray spectroscopy (EDS) mapping (Fig. 1(b)).

**Fig. 2:**
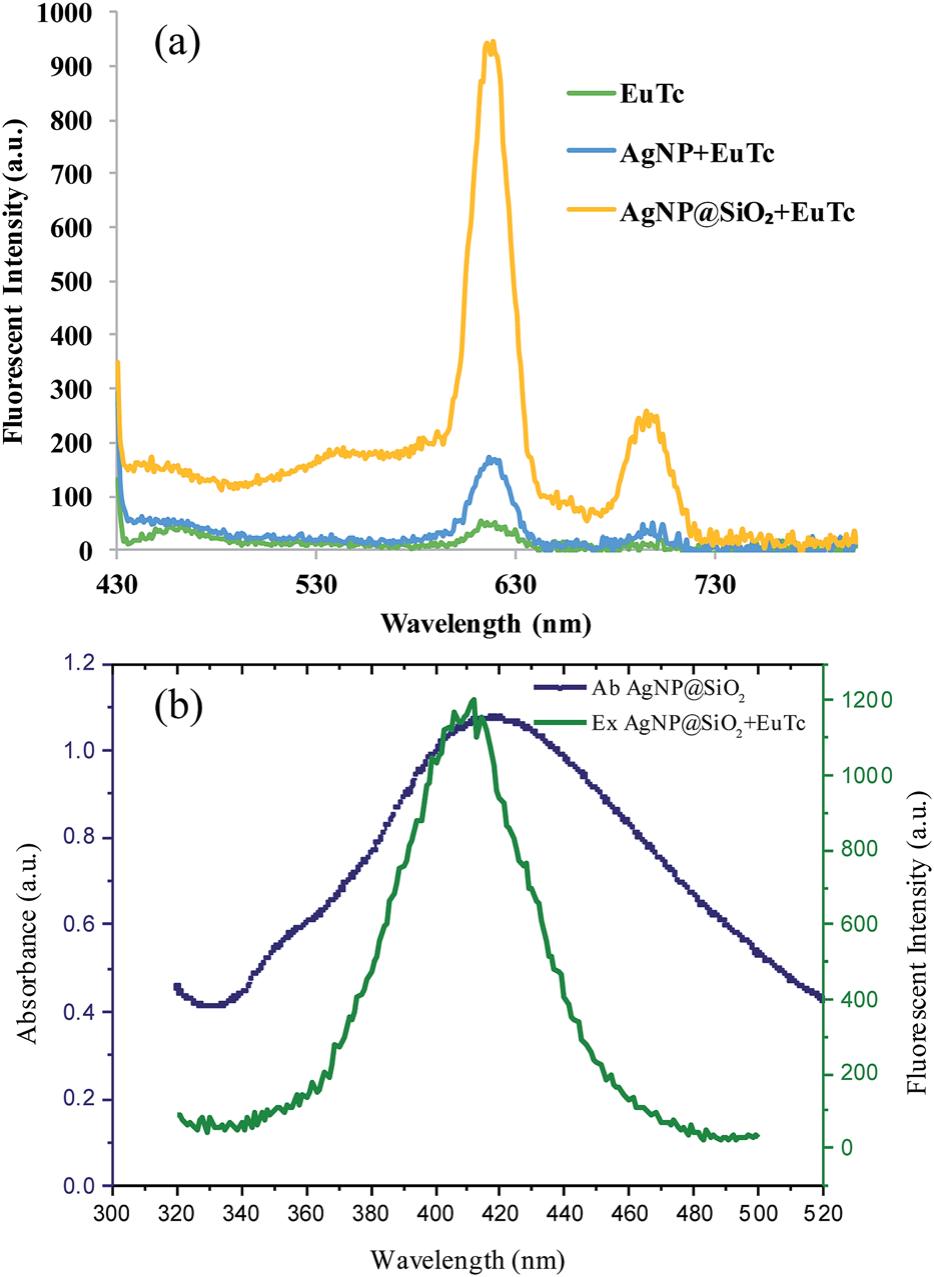
(a) Feasibility of the developed system for Tc. (b) Spectral overlap between the absorbance spectra of AgNP@SiO_2_ and the excitation spectra of EuTc-doped AgNP@SiO_2_. The excitation wavelength is set at 400 nm.

Next, we verified the feasibility of the developed strategy by measuring the fluorescence emission spectra from different samples. As illustrated in Fig. 2(a), the negligible fluorescence signal was generated from the EuTc complex (green curve) due to the fluorescence quenching by the coordinated water molecules,^24^ while the fluorescence intensity of the EuTc complex was enhanced in the presence of AgNP (blue curve), which is attributed to the replacement of the coordinated water molecules by the citrate ions on the surface of AgNPs and the MEF effect by AgNPs. However, the signal enhancement, which was defined as the fluorescence intensities of samples divided by fluorescence intensities of EuTc, was only 3-fold, which was because the distance between AgNPs and EuTc was too close to effectively induce the MEF effect. Most importantly, the maximum signal enhancement up to 18-fold was obtained when AgNP@SiO_2_ was added to (yellow curve), which provides the optimal distance between AgNP and EuTc complex for the effective MEF. It is also noteworthy that the absorbance spectra of AgNP@SiO_2_ overlapped with the excitation spectra of EuTc-doped AgNP@SiO_2_, which indicates that AgNP@SiO_2_ is suitable for the MEF effect to enhance the fluorescence signal of EuTc (Fig. 2(b)). In addition, AgNP@SiO_2_ with the different silica shell thickness from 13 nm to 44.36 nm (Table S1†) were prepared to find the optimal thickness for the effective MEF. As shown in Fig. S1,† when the shell thickness was around 30 nm, the highest fluorescence signal resulting from the most effective MEF was obtained. Overall, these results clearly confirmed that the silica shell on the surface of AgNP does not only provide a doping site for EuTc complex but also creates a proper distance for the MEF effect.^17^

**Fig. 3:**
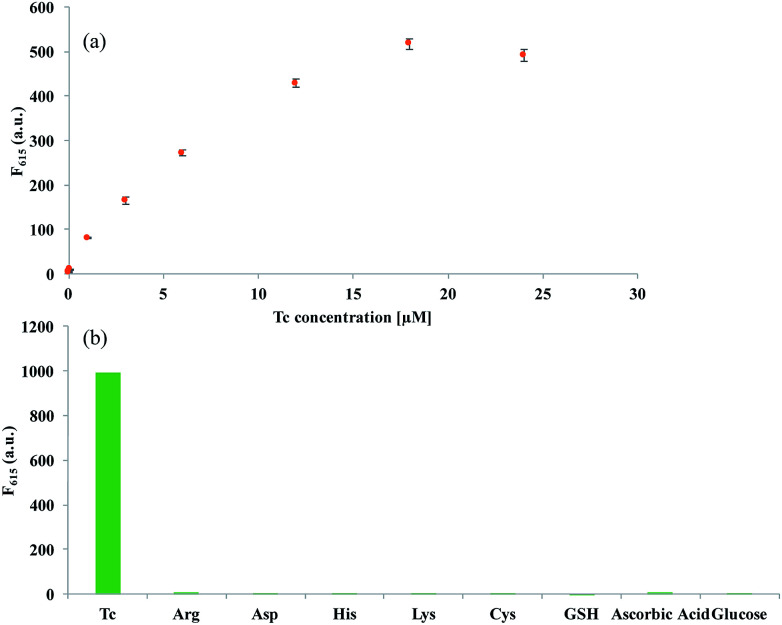
Sensitivity and selectivity of the developed system for Tc. (a) Fluorescent intensity at 615 nm (*F*_615_) of Eu^3+^-doped AgNP@SiO_2_ with various concentrations of Tc (0–24 μM). Insert: a linear relationship between *F*_615_ and the concentration of Tc (0–6 μM). (b) Fluorescent intensity at 615 nm (*F*_615_) of Eu^3+^-doped AgNP@SiO_2_ in the presence of Tc, amino acids, ascorbic acid, and glucose (the concentrations of Tc, amino acids, ascorbic acids, and glucose are all 10 μM). The excitation wavelength is set at 400 nm.

We then investigated the optimal conditions required for the efficient analysis of Tc. The results of experiments, in which the concentrations of Eu^3+^ (Fig. S2(a)†) and the AgNP@SiO_2_ (Fig. S2(b)†) were varied, demonstrate that 12 μM of Eu^3+^ and 0.02 nM of AgNP@SiO_2_, are ideal for the efficient analysis of Tc. Under the optimal conditions, we determined the detection sensitivity by measuring the fluorescent intensity at 615 nm (*F*_615_) as a function of Tc concentration. As shown in Fig. 3(a) and S3,† the fluorescence intensities increased with increasing concentrations of Tc up to 18 μM, but reached a plateau at concentrations higher than 18 μM. The plot of *F*_615_*vs.* Tc concentration shows that an excellent linear relationship is established in the range of 0–6 μM and the limit of detection (LOD) (3*σ*/slope) is 83.1 nM, which is comparable or better than the previous fluorescence-based methods.^25–27^

To evaluate the specificity of the present strategy towards Tc, the effect of the potential interfering species including amino acids, ascorbic acid, and glucose to induce the fluorescence enhancement was then examined. As shown in Fig. 3(b) and S4,† the strong fluorescence signal was observed only in the presence of Tc, but other interfering species led to negligible fluorescent signals, confirming the high selectivity of the developed system for Tc detection. Importantly, it should be noted that the developed system is quite simple and rapid with the signal-on fluorescence response, allowing the convenient and cost-effective determination of Tc.

It has been known that the fluorescence intensities of fluorescent dye or complex on the surface of AgNP@SiO_2_ become strongly enhanced only when the spectra between the fluorescent dye or complex and the localized surface plasmon resonance (LSPR) of the metal nanoparticles are overlapped. Thus, the fluorescence enhancement is highly dependent on the size and shape of metal nanoparticles that affects their LSPR.^28–31^

According to Mie Theory, the extinction of light for small nanoparticles is dominated by absorption, while for larger nanoparticles with the diameter over 50 nm, their extinction is dominated by scattering.^32–34^ Since MEF is the ability of the metal nanoparticle to scatter the coupled plasma, larger metal nanoparticles are regarded as the ideal materials for the effective MEF.^18^ In addition, it has been reported that almost all electrons in the nearly spherical AgNPs with the diameter of 10–50 nm experience the same phase of the incident electromagnetic field, which leads to the excitation of a dipole resonance.^35^ As the particle size increases over 50 nm, the excitation of higher plasmon modes such as a quadrupole resonance can be achieved in addition to the dipole resonance (Fig. S6 and S7†).^36^

In terms of the shape effect on the MEF, anisotropic nanoparticles such as silver nanoprisms and fractal-like structures, which provides the large scattering cross-section, have been known to enhance the local field more effectively than spherical nanoparticles.^14,28^ Specifically, the tips of the anisotropic nanoparticles can act as antennas, which effectively emit radiation when coupled to fluorophores. Thus, thicker metal structures with larger surface areas are preferred for the significant fluorescence enhancement through MEF.^37^

In addition, the silica shell thickness on the surface of AgNPs can also affect the MEF. In many reference papers, the silica shell thickness of 20–50 nm is found to be effective for the luminescence enhancement of the lanthanide complex and frequently utilized for the MEF.^38–40^ In principle, as the silica shell thickness increases over 20 nm, the radiative energy transfer becomes dominant as compared to the non-radiative energy transfer. Thus, 35 nm silica shell used in our work leads to the significant fluorescence enhancement through the radiative energy transfer from the excited AgNPs to the lanthanide complex (EuTc).^39^

Finally, the practical applicability of the developed strategy was verified by determining Tc in tap water. As shown in Fig. S5,† the fluorescence intensities in tap water increased as the concentration of Tc increased, which was almost comparable to that from the distilled water (Fig. 3), indicating that the proposed method is robust to the interfering substances present in the tap water. Then, the *F*_615_ from unknown samples were measured to determine the Tc concentration in tap water (Table 1). The results in Table 1 show that the reproducibility and precision of the developed method were quite good, yielding the Relative Standard Deviation (RSD) less than 5% and recovery rates of 89.3–105.9%. These observations clearly indicate that the developed strategy can be used to reliably determine Tc in real samples.

**Table 1:** Detection of Tc in tap water

Added Tc (μM)	Measured Tc^a^ (μM)	SD^b^	RSD^c^ (%)	Recovery^d^ (%)
1	1.058	21.045	1.988	105.883
3	3.037	131.425	4.326	101.267
6	5.359	36.451	0.680	89.324

a Mean of the three measurements.

b Standard deviation of three measurements.

c Relative Standard Deviation (RSD) = SD/mean × 100.

d Measured value/added value × 100.^41^

## Conclusions

In summary, we have developed a rapid, convenient and cost-effective method for the detection of Tc using the Eu^3+^-doped AgNP@SiO_2_ as a key detection component. By adjusting the shell thickness of AgNP@SiO_2_ that imparts the MEF effect, the most significant fluorescence enhancement in the presence of Tc was achieved, which was successfully utilized for the sensitive and selective determination of Tc. Importantly, this strategy does not require any expensive reagents or sophisticated instruments, which effectively overcomes the drawbacks such as the high cost and complexity in the previous Tc detection methods. Due to these outstanding features, the proposed MEF-based sensing method is expected to pave the way for the development of other chemical and biological sensing platforms.

## Conflicts of interest

There are no conflicts to declare.

## Supplementary Material

RA-008-C8RA03185A-s001
