# Supplementary material for: Development of a rapid and simple tetracycline detection system based on metal-enhanced fluorescence by europium-doped AgNP@SiO2 core–shell nanoparticles
Source: RSC Adv. 2018 Jul 5;8(43):24322–7. doi: 10.1039/c8ra03185a (PMC9082042; doi:10.1039/c8ra03185a)
Supplement: RA-008-C8RA03185A-s001 [file RA-008-C8RA03185A-s001.pdf]

Electronic Supplementary Material (ESI) for RSC Advances.
This journal is © The Royal Society of Chemistry 2018

Supporting Information

Development of a rapid and simple tetracycline detection system based on metal-enhanced fluorescence by europium-doped AgNP@SiO_2 core-shell nanoparticles

Pei Li[a], Sathish Kumar[a], Ki Soo Park[b,*], and Hyun Gyu Park[a,*]

[a]Department of Chemical and Biomolecular Engineering (BK 21+ program), KAIST, Daehak-ro 291, Yuseong-gu, Daejeon 34141, Republic of Korea

[b]Department of Biological Engineering, College of Engineering, Konkuk University, Seoul 05029, Republic of Korea

*To whom correspondence should be addressed.

Correspondence:

Hyun Gyu Park Ph.D., Department of Chemical and Biomolecular Engineering (BK21+ Program), KAIST, 291 Daehak-ro, Yuseong-gu, Daejeon 34141, Republic of Korea, Tel.: +82 42-350-3932, Fax: +82 42-350-3910, E-mail: hgpark@kaist.ac.kr

Ki Soo Park Ph.D., Department of Biological Engineering, College of Engineering, Konkuk University, Seoul 05029, Republic of Korea, Tel.: +82-2-450-3742, Fax: +82-2-450-3742, E-mail: kskonkuk@gmail.com

Tabel S1. Silica shell thickness of AgNP@SiO_2.

TEOS (mM)	Shell thickness (nm)	Standard deviation (nm)
0	0	-
1	13.00	8.12
2	34.28	5.59
3	33.83	5.17
4	44.36	7.49

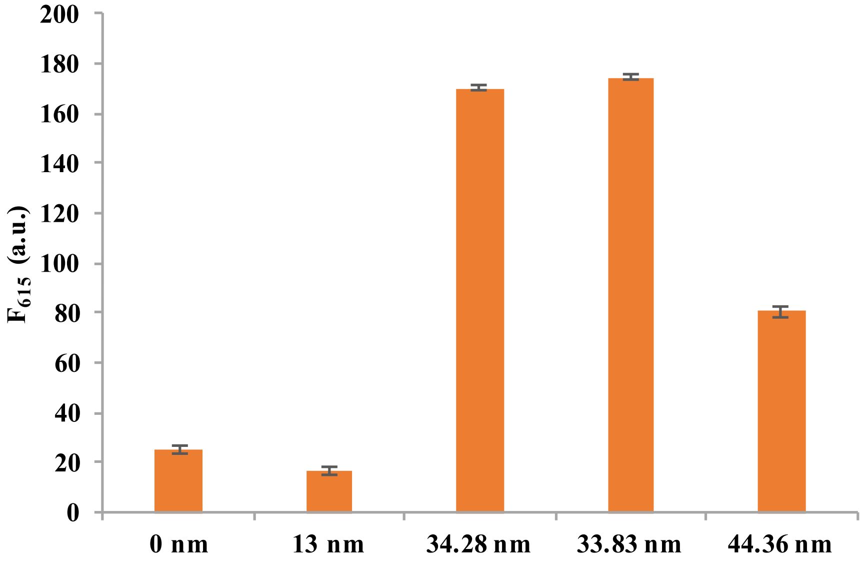

Figure S1. Fluorescent intensity at 615 nm (F_{615}) of EuTc-doped AgNP@SiO_2 with the different silica shell thickness. The excitation wavelength is set at 400 nm.

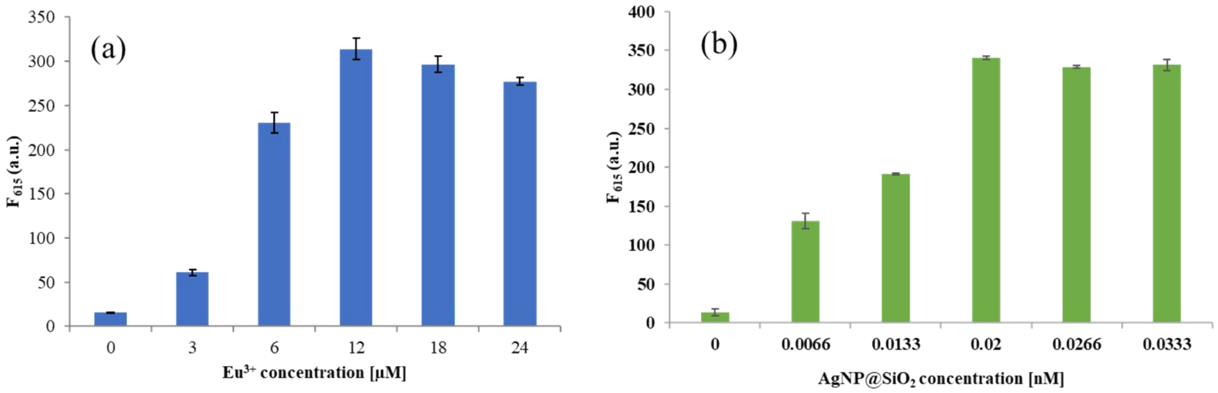

Figure S2. Optimization of (a) Eu^{3+} concentration and (b) AgNP@SiO_2 concentration. The excitation wavelength is set at 400 nm.

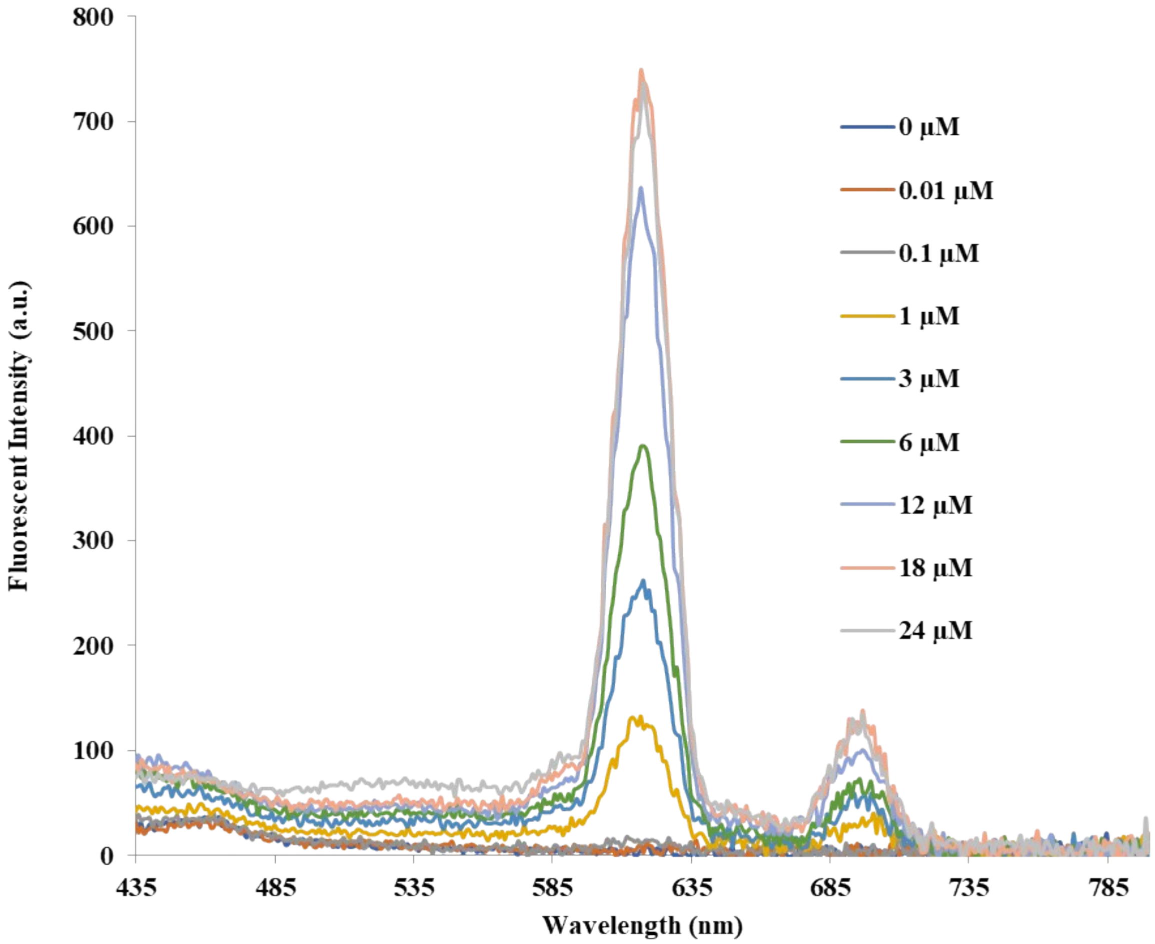

Figure S3. Fluorescence emission spectra of Eu^{3+}-AgNP@SiO_2 with various concentrations of Tc (0-24 µM). The excitation wavelength is set at 400 nm.

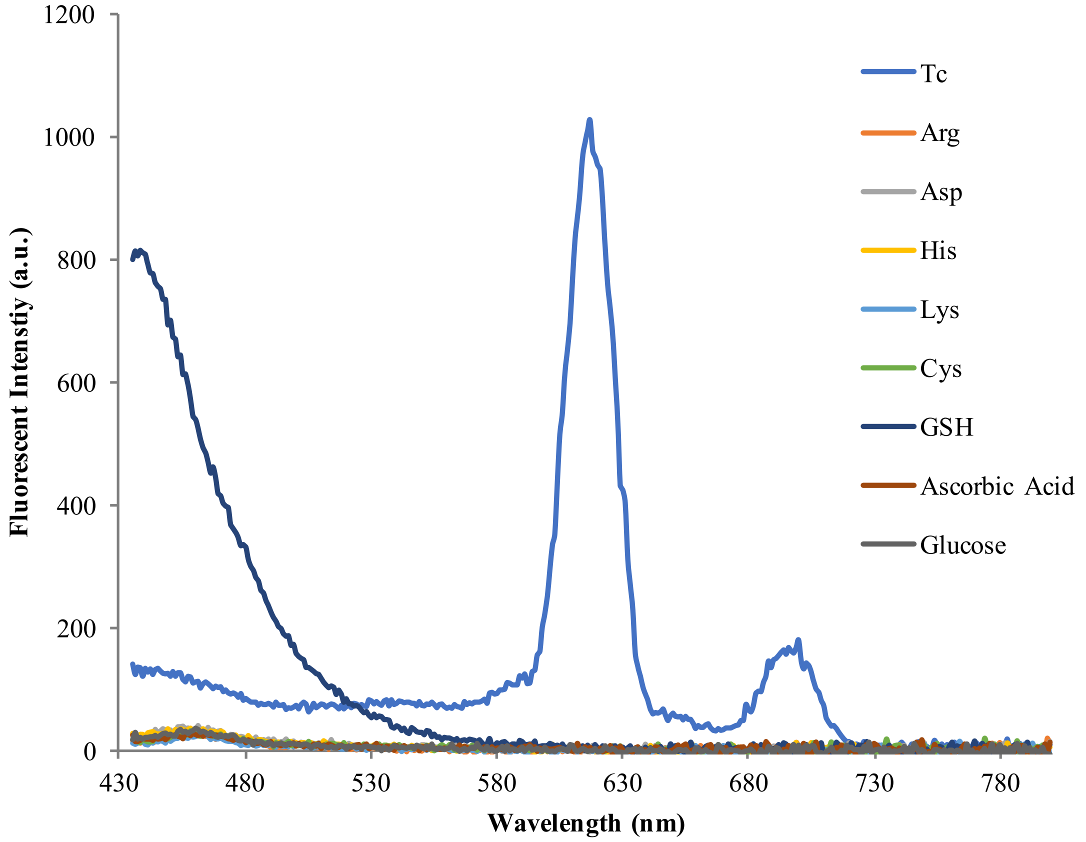

Figure S4. Emission spectra of Eu^{3+}-doped AgNP@SiO_2 in the presence of Tc, amino acids, ascorbic acid, and glucose. (The concentrations of Tc, amino acids, ascorbic acid and glucose are all 10 µM). The excitation wavelength is set at 400 nm.

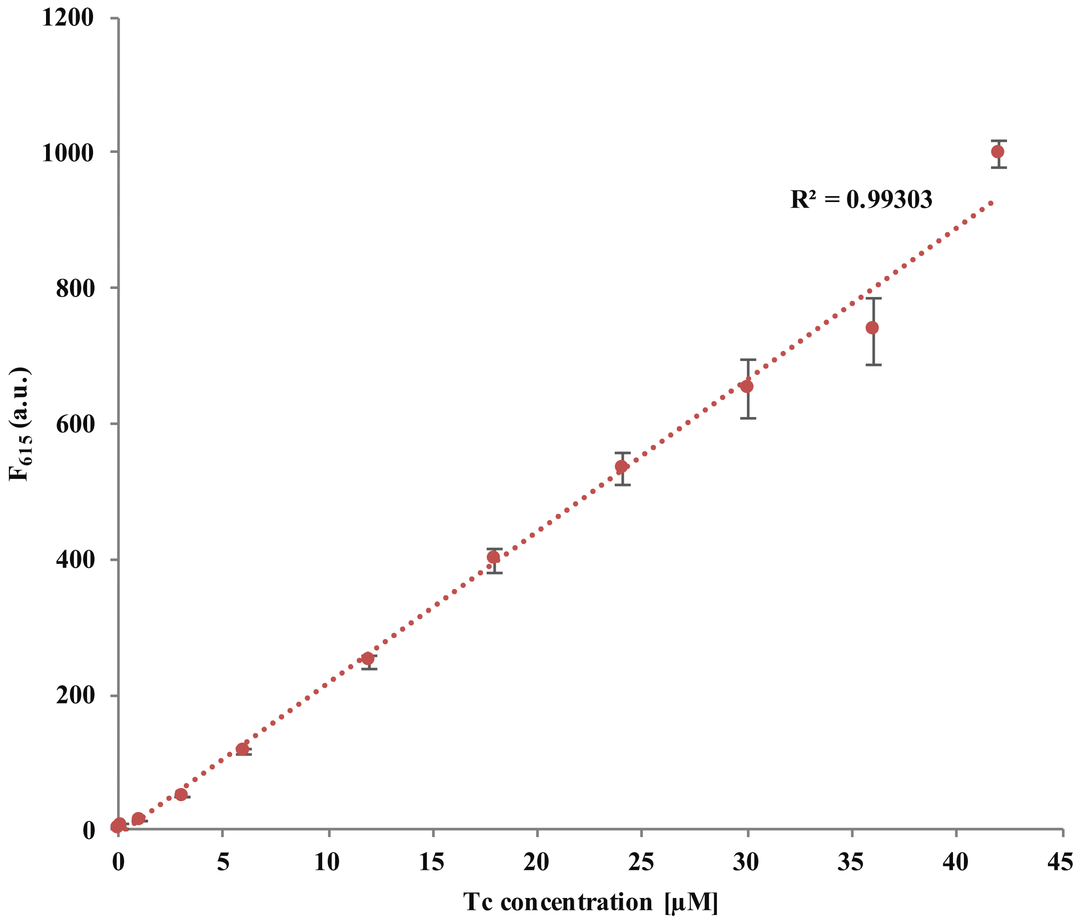

Figure S5. Detection of Tc in tap water.

We prepared small silver nanoparticles (AgNPs) with the average diameter of 5.29 nm in addition to the one with 51.51 nm (Figure S6), and compared the fluorescence intensities of EuTc in the presence of small and large AgNPs. As shown in Figure S7, in the presence of small AgNPs, the fluorescence intensities of EuTc was not enhanced, but quenched, while large AgNPs enhanced the fluorescence intensities up to 3-fold. This result is in accordance with the previous report, which explains that larger nanoparticles with a diameter of a few tens of nanometers are preferred for the effective MEF than small nanoparticles. [1]

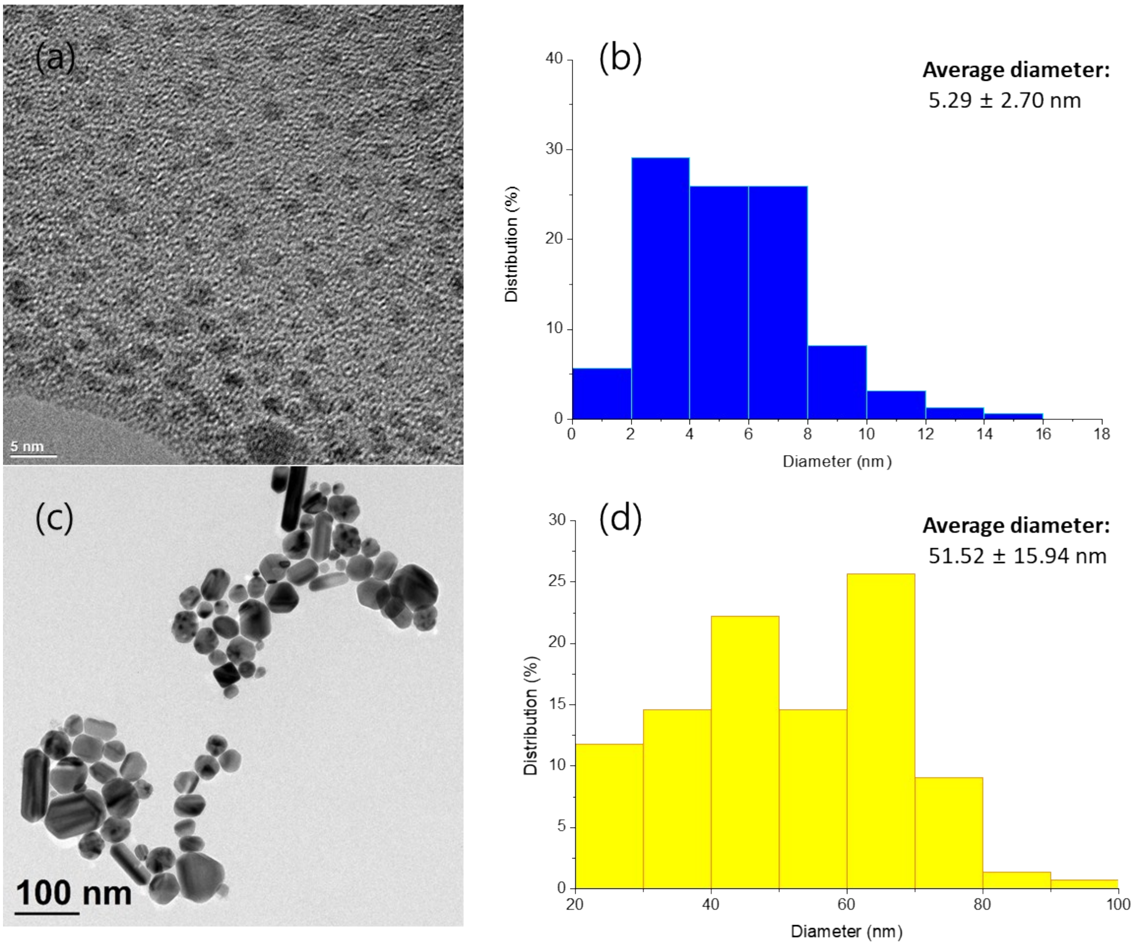

Figure S6. TEM image and size distribution of small (a and b) and large (c and d) AgNPs**.**

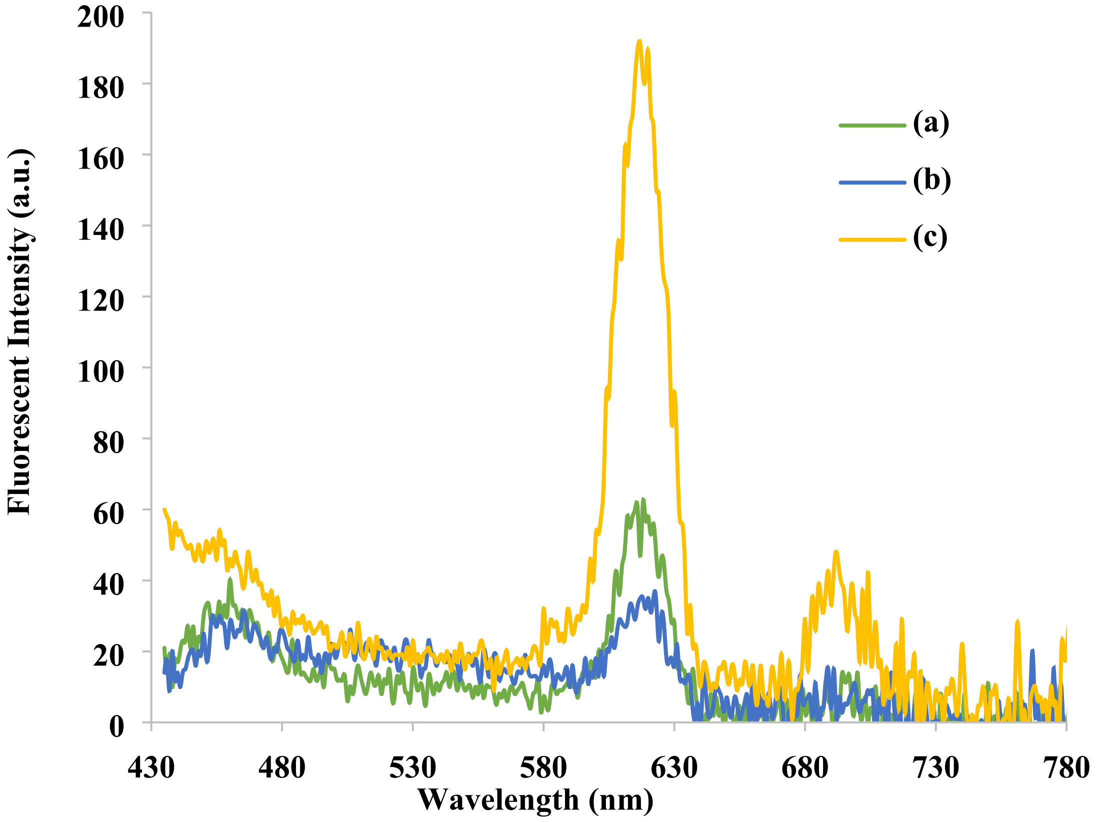

Figure S7. Fluorescence intensity of (a) EuTc, (b) EuTc doped-small AgNPs and (c) EuTc doped-large AgNPs.

References and notes

1. C. D. Geddes, *Metal-enhanced fluorescence*, John Wiley & Sons, 2010.
